# HERON: High-Efficiency Real-Time Motion Quantification and Re-Acquisition for Fetal Diffusion MRI

**DOI:** 10.1109/TMI.2025.3569853

**Published:** 2025-05-14

**Authors:** Jordina Aviles Verdera, Antonia Bortolazzi, Sara Neves Silva, Kelly Payette, Kamilah St. Clair, Sarah McElroy, Shaihan Malik, Joseph V Hajnal, Raphael Tomi-Tricot, Mary A Rutherford, Jana Hutter

**Affiliations:** Imaging Physics and Engineering Research DepartmentSchool of Biomedical Engineering and Imaging SciencesKing’s College London (KCL) SE1 7EH London U.K.; Early Life Imaging DepartmentSchool of Biomedical Engineering and Imaging SciencesKing’s College London (KCL) SE1 7EH London U.K.; Smart Imaging Laboratory, Radiologisches InstitutUniversitätsklinikum Erlangen Erlangen 91054 Germany; MR Research Collaborations, Siemens Healthcare Ltd. GU16 8QD Camberley U.K.

**Keywords:** Fetal MRI, diffusion, motion correction, AI

## Abstract

Fetal diffusion MRI (dMRI) provides fascinating and clinically crucial insights into the microstructure of the human brain during development, but is highly sensitive to motion artifacts of fetal movement and maternal breathing, which impact data quality and limit diagnostic accuracy. This study introduces HERON, a robust, real-time, automatic pipeline designed to enhance fetal brain dMRI by performing motion assessment and re-acquisition. HERON leverages AI-driven brain localization, segmentation, and motion assessment on a clinical 0.55T scanner to automatically plan, quality check, and reacquire motion-affected dMRI volumes. Remaining inter-volume motion is corrected during post-processing. Tested in 20 cases, the pipeline effectively improved image quality, reduced intra- and inter-volume motion, and enabled more reliable quantitative analysis even in challenging cases. Excellent agreement with human observers (specificity 97%, sensitivity 92%) was shown and the mean Apparent Diffusion Coefficient and Intravoxel Incoherent Motion dropped in the majority of cases after correction. Improving fetal dMRI through an automatic AI-driven pipeline enables higher diagnostic quality and thus potentially wider use in both research and clinical applications.

## I. Introduction

Diffusion MRI (dMRI) offers fascinating insights into early human brain development by visualizing and quantifying the maturation of the micro-architecture. It is particularly well suited for assessing white matter maturation and mapping early connectivity patterns, which are fundamental to understanding normal and abnormal neural development [1], [2], [3]. Furthermore, it plays a key role in clinical management as it allows precise imaging of crucial structures, such as the corpus callosum - a major white matter tract affected by conditions such as Agenesis of the Corpus Callosum (ACC) or Corpus Callosum Hypoplasia (CCH) - which is essential for accurate diagnosis and assessment [4], [5]. Finally, fetal dMRI has also been applied outside the brain, clinically, for example, in the antenatal diagnosis of renal disease [6], and in research settings in the placenta [7] or the fetal lung, among others [8].

Most commonly and particularly widespread in fetal imaging, dMRI data is acquired with a single shot diffusion-weighted spin-echo echo planar imaging (DW-SE-EPI) sequence. A pair of opposed gradients, parametrized by strength (b-value) and direction (b-vector), are placed on either side of the refocusing pulse, followed by a fast EPI read-out. This is repeated for each 2D slice to obtain 3D volumes of the region of interest. The process is repeated for a range of b-vectors and b-values, depending on the planned analysis, ranging from single direction protocols with a limited number of b-values to comprehensive acquisitions with a large range of b-values, to enable, for example, intra-voxel incoherent motion (IVIM) analysis [9], or with a large range of b-vectors (e.g. High-Angular Resolution (HARDI) schemes) to enable tractography [10]. These more advanced schemes offer critical insights into processes such as subplate development [11], [12], early white matter organization [10], [13], presence of fiber bundles [14] and tract maturation [15], [16] during this dynamic phase of brain growth.

However, the challenges present in all fetal MRI examinations such as involuntary fetal motion, air-tissue interfaces created by maternal anatomy, as well as the substantial variability inherent to fetal development, are exacerbated in dMRI [17], [18] by the requirement of voxel-specific analysis on data acquired over a couple of minutes. Thus, accurate results for clinical and research applications depend on motion detection and correction.

Recent advances have significantly improved the reliability and quality of quantitative fetal MRI, particularly through the integration of artificial intelligence (AI) for motion characterization, correction, tracking and quality control, primarily during post-processing [19], [20], [21], [22], [23]. Furthermore, the incorporation of these advanced techniques into the acquisition phase allows real-time responsiveness and improves the efficiency of the acquisition [24].

In adult brain dMRI, patient motion - whether voluntary or involuntary - can also significantly compromise image quality and hinder accurate analysis. Retrospective techniques, such as image registration and advanced reconstruction algorithms, have been widely utilized to correct motion-induced corruption after acquisition [25], [26], [27], [28], [29]. More recently, the development of advanced motion detection technologies has paved the way for minimizing motion artifacts during acquisition itself. Techniques such as prospective motion correction and real-time feedback mechanisms, using external devices [30], navigators [31], optical marker-less motion tracking [32], phase image-based methods [33], and combined magnitude and phase image-based methods [34], have demonstrated significant promise in reducing artifacts and improving both image quality and quantitative analysis.

The unique challenges associated with fetal imaging specified above hinder the direct application of these prospective and real-time dMRI motion correction techniques. This is especially relevant for the increasingly sophisticated models applied to study the fetal period. Such advancements increase the requirement to address motion-related artifacts to ensure accurate analysis. Taking into account all these obstacles, our study aims to explore novel approaches to integrate real-time motion information into fetal brain dMRI during acquisition, with the goal of improving image quality, acquisition efficiency, and quantitative analysis.

Recent advances have highlighted the potential of automatic segmentation and quality assessment pipelines applied to multi-echo diffusion-weighted fetal data on low-field systems, achieving high-quality fetal brain segmentations and delivering precise and informative quality and motion metrics [35]. Expanding on this foundation, we present HERON (High-Efficiency Real-time mOtion quantification and re-acquisitioN) - an automated, real-time, image-based pipeline that performs automatic axial fetal brain planning, fetal brain segmentation, intra- and inter-volume motion assessment, and volume quality control - allowing real-time re-acquisition of corrupted volumes and paving a way towards widespread robust usage of fetal dMRI.

## II. Methods

### A. HERON Pipeline

**Fig. 1.**
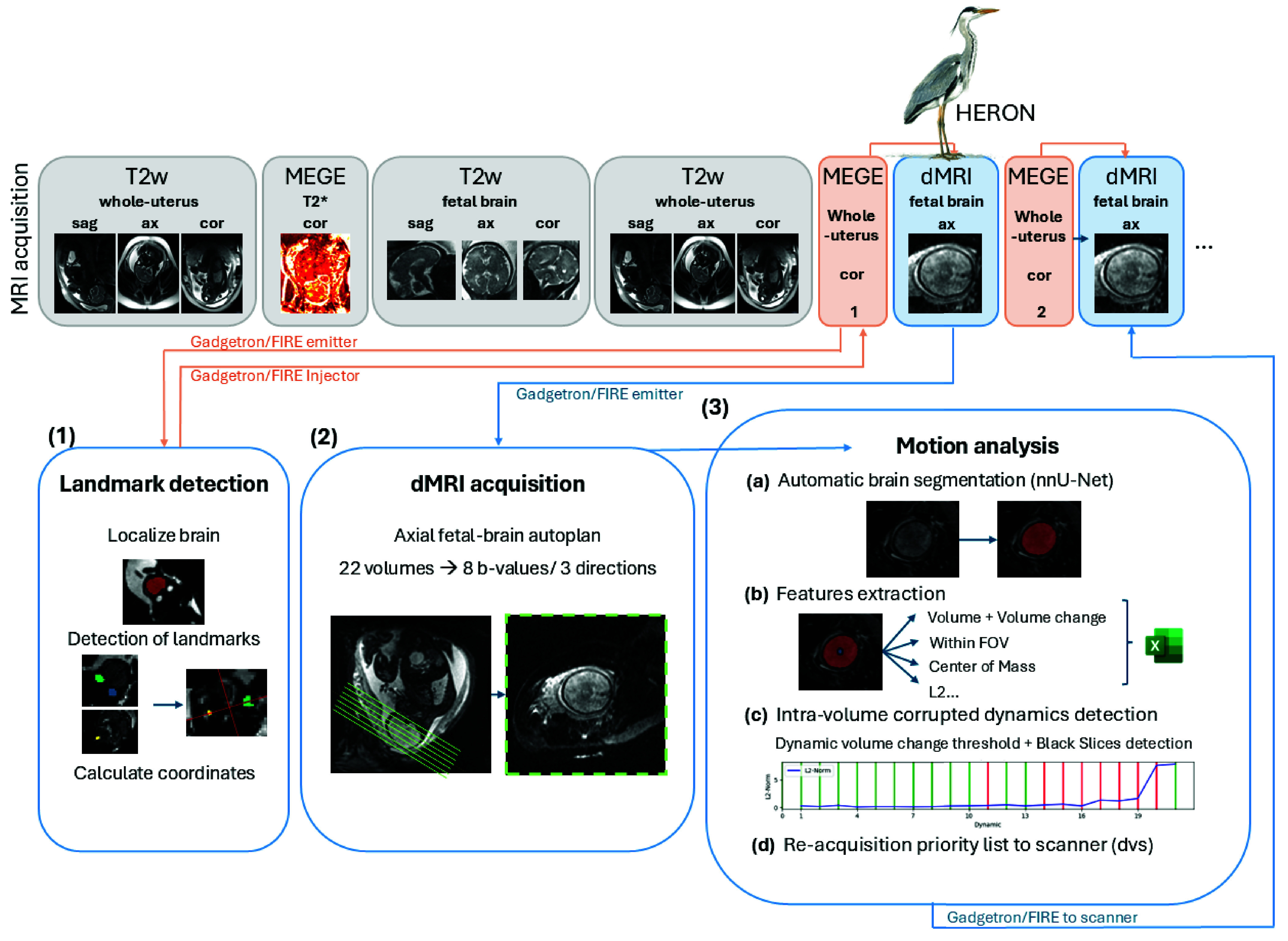
Pipeline illustrating the proposed real-time fetal diffusion package - including (1) a multi-echo gradient echo scan for automatic landmark detection and planning, (2) the diffusion MRI scan with the prescribed bval/bvec set, followed by (3) automatic motion analysis including (a) automatic brain segmentation, (b) motion features extraction, (c) intra-volume motion detection and generation of a prioritized list for the final diffusion scan, (d) reacquiring the volumes with the highest intra-volume motion.

The complete proposed prospective pipeline is graphically depicted in Fig. 1. It is composed of three main steps: (1) Key landmarks are identified on a short gradient echo sequence and used to automatically plan the diffusion scan. (2) The diffusion data acquisition is acquired. (3) A real-time quality assessment is performed to generate a prioritized list of corrupted volumes and these are then reacquired. The framework was implemented on a 0.55T MAGNETOM Free.Max scanner (Siemens Healthineers, Forchheim, Germany), equipped with the FIRE tool [36] and the Gadgetron framework [37] and connected to a high performance computer (Intel i9-10920X (3.5GHz) 19.25MB Cache, RAM 128GB Corsair VENGEANCE DDR4 3000MHz ( $8 \times 16$GB), 11GB NVIDIA GEFORCE RTX 2080 Ti).

For step (1), a short multi-echo gradient echo sequence is acquired in the maternal coronal plane covering the entire uterus. The resulting images are processed with a network to localize the brain and identify specific landmarks (computation time 9 sec). The coordinates of the landmarks are written to a text file, and read in by the dMRI sequence to enable fully automatic planning of the axial radiological plane.

In step (2), the dMRI data sets are acquired with a single shot DW-SE-EPI sequence that has been adapted to automatically calculate the position of the axial radiological brain planes based on the previously identified landmarks contained in the text file [24]. The sequence parameters were as follows: TR = 7200 ms, TE = 129 ms, TA = 2.43 min, FOV $ = 400 \times 400$ mm, 35 slices, echo spacing 0.97 ms, no fat saturation, resolution = 3 mm^3^, partial Fourier = 6/8. 7 b-values = [10, 50, 80, 200, 400, 600, 1000] s/mm^2^ in addition to b=0s/mm^2^ were chosen with (b-values >0 s/mm^2^ acquired in three orthogonal diffusion directions).

Finally, in step (3), the quality checks and the priority list for reacquisition are obtained through four different steps (3a-3d) individually described in the following paragraphs.

In step (3a), an automatic segmentation of the fetal brain is obtained for each acquired volume (b-vector/b-value combination). This automatic real-time segmentation is enabled by a 3D network trained in-house based on the nn-UNet framework [38]. To enhance robustness and ensure broad applicability, the network was trained and validated on a data set of 2989 fetal scans, incorporating variations across three field strengths (0.55T, 1.5T, and 3T), two vendors (Siemens and Philips), and five different contrasts (single-shot fast spin echo, balanced Steady-state free precession, dMRI, T1 and T2* maps). The segmentation is obtained in approx. 40 sec for all volumes performing 5-fold cross-validation, or roughly 1.8 sec per volume.

In step (3b), a comprehensive set of inter-volume and intra-volume quality and motion analysis related features are derived from these segmentations. The brain volume is extracted from the segmentation, followed by the calculation of the position within the field of view (FOV) and the brain’s center of mass (COM). For the evaluation of the motion between volumes, the average Euclidean distance (L2 norm) between the brain COM positions is calculated between consecutive time points. This metric quantifies the spatial shift of the brain, thus providing a measure of motion between adjacent volumes. In addition, HERON monitors whether the total volume of the brain remained consistently within the FOV during each acquisition by assessing the fraction of non-zero voxels touching the FOV boundaries. This step helps to capture potential cropped volumes.

In step (3c), the checks for intra-volume motion detection are performed with two metrics. The mean intensity of each slice (to detect motion/motion-induced signal loss) and changes in brain volume (through-plane) are calculated. To detect motion-induced signal loss, the mean intensity of each slice is computed and compared to the mean intensity of the entire volume of the brain. If the mean intensity of a slice is 35% lower than the mean volumetric intensity, it is classified as corrupted. This threshold was selected as a balance between motion-induced signal loss and naturally lower signal intensities in certain brain structures, such as the cortical boundaries or ventricles, which exhibit lower signal values under normal conditions. To detect through-plane motion, volume changes are compared to the volume from the first dynamic. A thresholding approach taking the inherent attenuation related to the applied b-value into account is chosen. The so-determined threshold is applied to the volumes obtained from the segmentations. For generalizibility, the threshold is modeled as a scaled function of the b-value and the apparent diffusion coefficient (ADC):

\begin{equation*} \textrm {Threshold}_{b} = \alpha \times (1 - e^{-b \times ADC})+ f \tag {1}\end{equation*}

The additional factor f was chosen as $f=0.02$ to allow a fixed segmentation error of 2% for all volumes, the scaling factor $\alpha = 0.3$ was empirically optimized to strike a balance between sensitivity to biologically significant changes and robustness to noise. Considering that cerebrospinal fluid (CSF) surrounds the fetal brain and exhibits high diffusivity, we set the ADC to 0.002 mm2/s. As the b-values increase, the signal from CSF attenuates rapidly, which can affect the network’s ability to accurately segment the volume. This approach allows for the detection of meaningful diffusion-related changes minimizing false positives, especially at high b-values. Corrupted volumes are identified as those that contain any slices with excessive signal loss (e.g. black slices) or which exhibit a volume change that exceeds a b-value-dependent threshold.

As the final step (3d), a file containing the b-values and b-vectors of the identified corrupted volumes is created and sent to the scanner. These volumes are re-acquired and replaced in their corresponding dynamic in the main acquisition. This process can be repeated as needed, facilitating iterative refinement of data quality while considering acquisition time constraints. In this study, re-acquisitions were limited to a maximum of two iterations. Total computation time for steps 3b, 3c and 3d is approximately 10 sec.

### B. Experiments, Validation and Analysis

The fetal dMRI data included in this study was acquired on a 0.55T clinical scanner (MAGNETOM Free.Max, Siemens Healthineers, Forchheim, Germany) for a total of 20 participants with the entire HERON pipeline as described applied in real time. Nine of the presented cases used an earlier version of the pipeline, which relied on a fixed threshold to detect volume changes, in contrast to the dynamic thresholding approach implemented in the presented HERON pipeline. The dynamic thresholding described here was retrospectively applied to these cases for comparative evaluation. A post-processing step was applied to correct inter-volume motion artifacts using the mrtrix3 framework [39], including image denoising and rigid volume registration.

For validation of the segmentation network as a first crucial step in HERON, the performance of the trained network was evaluated using Dice Scores on an independent test set, composed of 299 images from 149 different participants, encompassing a range of contrast, gestational ages, vendors and field strengths, manually segmented by experienced researchers in the field of fetal MRI.

To validate the detection of corrupted volumes, for all subjects, the motion-corrupted volumes within the respective first acquisition were visually identified by an expert fetal imaging researcher, with these results compared to volumes automatically flagged as corrupted. Furthermore, to validate the choice of $\alpha $, the differences between excluded volumes based on the expert annotation and the automatic algorithm with values varying from 0.1 to 0.5 were evaluated.

Diffusion analysis was performed using the dmipy toolkit [40], applying both a monoexponential model to estimate ADC and a more advanced IVIM model to generate diffusivity, pseudo-diffusivity and fraction maps. The residuals from the model fits were saved for subsequent analysis. Furthermore, to evaluate the changes quantitatively, the mean ADC and IVIM values as well as the standard deviation were measured inside two regions-of-interest (ROIs), annotated by an expert in fetal imaging within the deep grey matter and the temporal white matter.

## III. Results

HERON was successfully applied prospectively in a total of 20 cases. It was able to detect in real-time that the scan was motion-free in 3 cases, hence not triggering reacquisition, 9 cases were successfully identified and corrected through reacquisition, 3 cases retained uncorrected volumes after two re-acquisitions, 1 case had excessive motion and 4 cases were discarded due to presence of artifacts or full brain not included in the FOV.

**Fig. 2.**
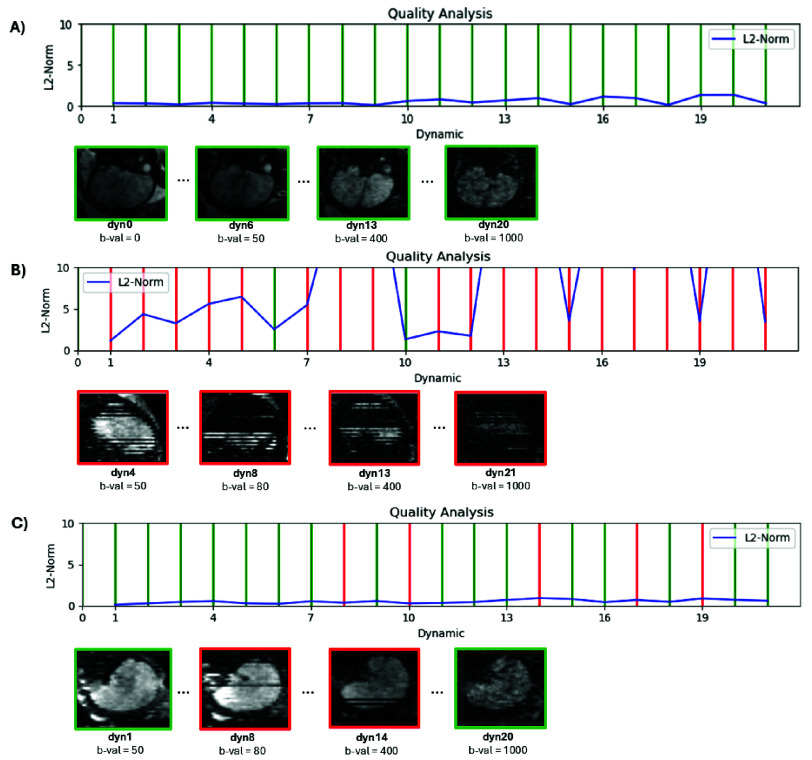
Complete real-time assessment in 3 exemplary cases, displaying respectively the line plots and four selected volumes in reformatted sagittal view, for motion-free in green and motion-corrupted volumes in red.: (A) no significant motion identified, (B) high degrees of motion and (C) moderate motion.

Exemplary results for three different motion scenarios - no motion, high motion, and low motion - are presented in Figure 2. Line plots depict the inter-volume motion represented by average L2 scores, along with the results of the intra-volume motion assessment indicated by green (motion-free) and red (motion-corrupted) lines. For each scenario, examples of volumes with different quality scores are provided to illustrate the differences in image quality caused by motion artifacts. (A) represents a case where all volumes were classified green with respect to intra-volume motion and show a low degree of inter-volume motion (L2-Norm <2 cm for all volumes), (B) shows a high degree of inter-volume motion with the L2-Norm >10 cm for eight volumes, and most volumes were ranked “red” for the intra-volume score, corresponding well to the depicted volumes, showing in the sagittal reformatted views significant signal drop and staircase artifacts. Finally, (C), shows a case with low inter-volume motion (L2-Norm <2). Five volumes were identified as ‘red’ (indicating high intra-volume motion) and 17 as ‘green’ (indicating low intra-volume motion). Two examples from each category are shown, illustrating both uncorrupted volumes and corrupted ones.

**Fig. 3.**
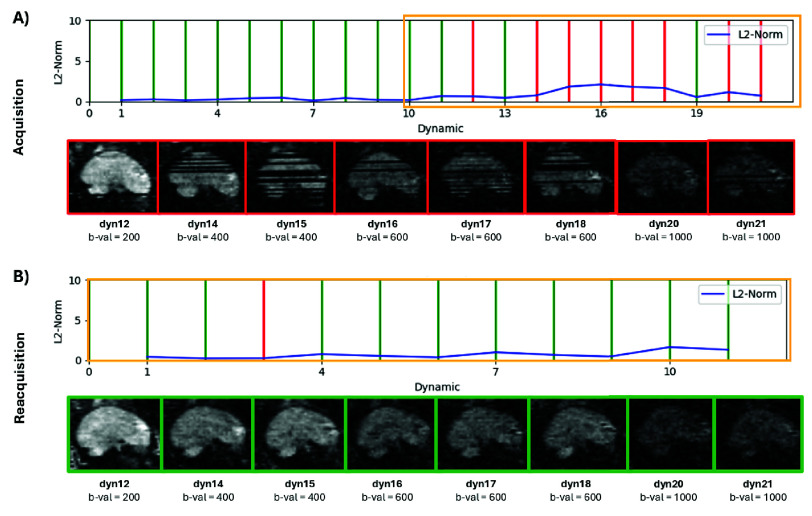
Real-time results from the re-acquisition in a moderate motion case, illustrating (A) The initial acquisition line plot and the correspondent 8 corrupted volumes spanning 4 b-values (yellow rectangle) and (B) line plot for the re-acquisition with a total of 5 b-values, correcting all the previously affected dynamics. Green depicts motion-free, red motion-corrupted.

Figure 3 provides a more detailed analysis of the acquisition and reacquisition process for a case with moderate motion. Figure 3A shows the identification of a series of motion-corrupted volumes, highlighted by the yellow box, in the second half of the original acquisition. The volumes labeled “red” are displayed in the sagittal, reformatted views underneath and show clear dropout slices and artifacts. Figure 3B shows the quality of these volumes improved greatly during the reacquisition.

**Fig. 4.**
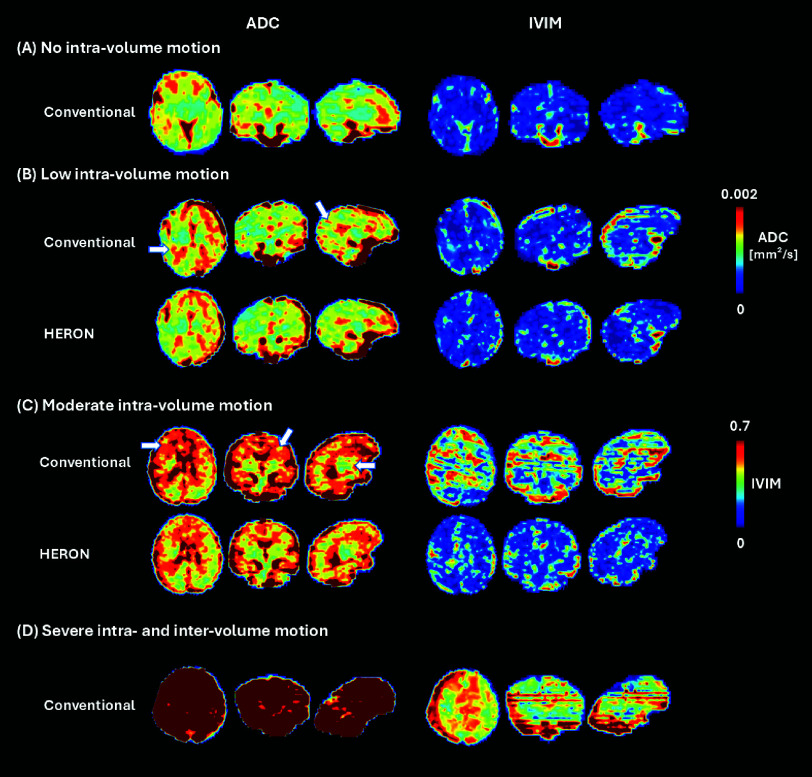
Apparent diffusion coefficient and intra-voxel incoherent motion tissue fraction map maps obtained for four different cases: (A) case with no intra-volume motion, (B) low motion case pre and post reacquisition, (C) moderate motion case pre and post reacquisition and (D) case with high intra- and inter-volume motion, in reformatted axial, coronal and sagittal orientations. The arrows indicate areas of image artifacts.

**Fig. 5.**
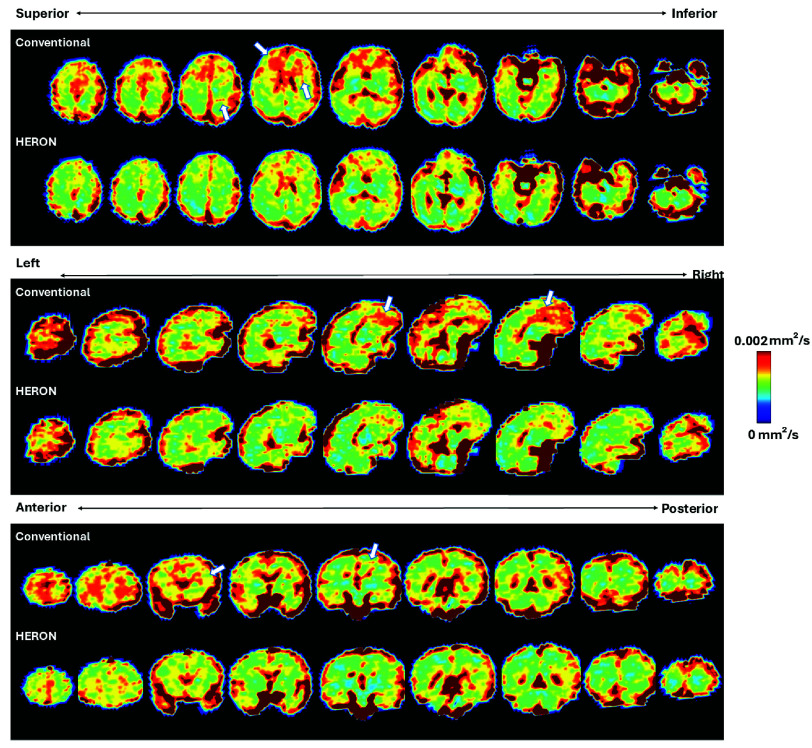
Whole brain ADC map results in all three radiological orientations before and after the application of the HERON pipeline in a moderate motion case. The arrows indicate areas of image artifacts.

Figure 4 presents the ADC and IVIM maps for four cases with varying levels of motion, highlighting the critical role of reacquisition to address motion that otherwise leads to inaccurate qualitative measures such as details visible in the frontal lobe and cortex in C, pointed with a white arrow. The results demonstrate the effectiveness of the pipeline in mitigating motion-induced artifacts and preserving fine structural details, ensuring higher-quality quantitative results. This is further illustrated in Figure 5, showing randomly selected ADC map slices across the volume in three different fetal brain radiological planes in a case with moderate motion (5 corrupted volumes) for both the conventional acquisition and the HERON post-processed reacquisition.

**Fig. 6.**
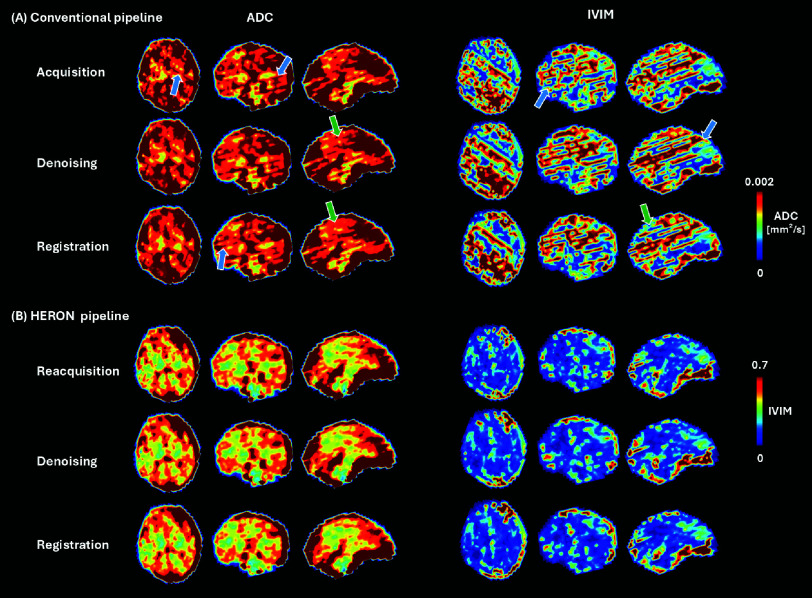
Whole brain apparent diffusion coefficient and intra-voxel incoherent motion tissue fraction map maps in a moderate motion case obtained with the conventional pipeline (no reacquisition) and the HERON pipeline at three different stages: no post-processing, after denoising and denoising plus registration. Typical stripe artifacts originating from black out slices are indicated with blue arrows and motion-artifacts pre-and post registration with green arrows.

Figure 6 presents the ADC and IVIM maps at different stages of the quantitative analysis for a moderate motion case (8 corrupted volumes): no post-processing, denoising and a combination of denoising and registration for a moderate motion case (8 corrupted volumes). The major difference is apparent between A and B, indicating for example a deep gray matter region with a pink arrow highlighting the qualitatively improved anatomical impression, a blue arrow the reduction in stripe artifacts and a green arrow the decrease in ADC in the frontal lobe. The differences within the steps in A and B respectively are qualitatively minor.

The validation analysis in Figure 8 demonstrated an agreement of 96.3% between manual and automatic intra-volume quality assessments with a specificity of 97% and a sensitivity of 92%, highlighting the precision of the HERON pipeline in detecting motion-corrupted volumes. Discrepancies primarily arose from two factors: (i) inter-volume changes that, despite not corrupting the volume, result in subtle volume changes misclassified as corrupted by HERON’s dynamic thresholding and (ii) a slightly rotated axial plane resulting in oblique cuts of the longitudinal fissure. Visually, this changed angle leads to an apparent wider CSF space which could result in a slice misclassified as a blackout slice. The ROI analysis in table I shows a decrease in ADC and IVIM after correction in 5 out of 9 cases, visually depicted also in 2 examples in 7. The quantitative evaluation for different values of $\alpha $ shown in Figure 9 plateaus between 0.3 and 0.4, resulting in the choice of 0.3 to ensure further robustness. The network validation experiment showed a Dice Score for the test set of $0.925 \; \pm \; 0.058$ when evaluating fetal brain segmentation accuracy.

**TABLE I:** Regional Quantitative Results of Mean Apparent Diffusion Coefficient (ADC) and Intravoxel Incoherent Motion (IVIM) for White and Gray Matter in the Cases Corrected by HERON, Comparing the Conventional Pipeline With HERON Results

		Conventional ADC [mm2/s]	HERON ADC [mm2/s]	Conventional IVIM	HERON IVIM	No. corrupted volumes
Case 1	Mean WM	0.00202 +/- 0.00030	0.00159 +/- 0.00031	0.470 +/- 0.26	0.239 +/- 0.12	7
	Mean GM	0.00158 +/- 0.00031	0.00115 +/- 0.00025	0.379 +/- 0.26	0.114 +/- 0.07	
Case 2	Mean WM	0.00149 +/- 0.00019	0.00145 +/- 0.00021	0.124 +/- 0.07	0.097 +/- 0.08	5
	Mean GM	0.00105 +/- 0.00021	0.00106 +/- 0.00019	0.088 +/- 0.05	0.093 +/- 0.10	
Case 3	Mean WM	0.00120 +/- 0.00012	0.00113 +/- 0.00013	0.107 +/- 0.13	0.026 +/- 0.03	4
	Mean GM	0.00109 +/- 0.00062	0.00106 +/- 0.00051	0.091 +/- 0.10	0.084 +/- 0.12	
Case 4	Mean WM	0.00138 +/- 0.00020	0.00136 +/- 0.00020	0.112 +/- 0.13	0.097 +/- 0.12	3
	Mean GM	0.00119 +/- 0.00018	0.00115 +/- 0.00016	0.114 +/- 0.08	0.081 +/- 0.05	
Case 5	Mean WM	0.00152 +/- 0.00020	0.00131 +/- 0.00019	0.307 +/- 0.24	0.127 +/- 0.10	5
	Mean GM	0.00122 +/- 0.00031	0.00111 +/- 0.00017	0.220 +/- 0.22	0.061 +/- 0.03	
Case 6	Mean WM	0.00141 +/- 0.00019	0.00140 +/- 0.00019	0.055 +/- 0.07	0.056 +/- 0.07	1
	Mean GM	0.00110 +/- 0.00021	0.00110 +/- 0.00021	0.062 +/- 0.09	0.063 +/- 0.09	
Case 7	Mean WM	0.00131 +/- 0.00020	0.00123 +/- 0.00015	0.028 +/- 0.04	0.054 +/- 0.08	1
	Mean GM	0.00093 +/- 0.00019	0.00094 +/- 0.00019	0.025 +/- 0.03	0.037 +/- 0.05	
Case 8	Mean WM	0.00126 +/- 0.00017	0.00127 +/- 0.00017	0.054 +/- 0.06	0.053 +/- 0.04	3
	Mean GM	0.00098 +/- 0.00033	0.00096 +/- 0.00030	0.036 +/- 0.06	0.033 +/- 0.05	
Case 9	Mean WM	0.00160 +/- 0.00019	0.00160 +/- 0.00017	0.127 +/- 0.12	0.120 +/- 0.11	3
	Mean GM	0.00131 +/- 0.00018	0.00131 +/- 0.00020	0.105 +/- 0.09	0.103 +/- 0.08

**Fig. 7.**
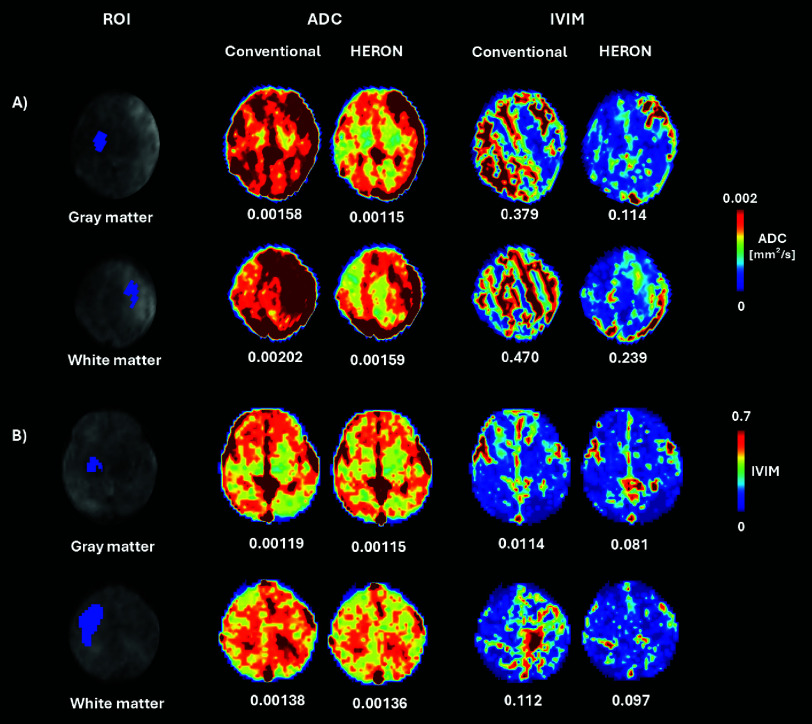
Quantitative results showing manual segmentation of the regions of interest together with the Apparent Diffusion Coefficient(ADC) and the Intravoxel Incoherent Motion (IVIM) maps comparing the conventional pipeline with HERON results in a case with (A) high motion and a case with (B) moderate motion.

**Fig. 8.**
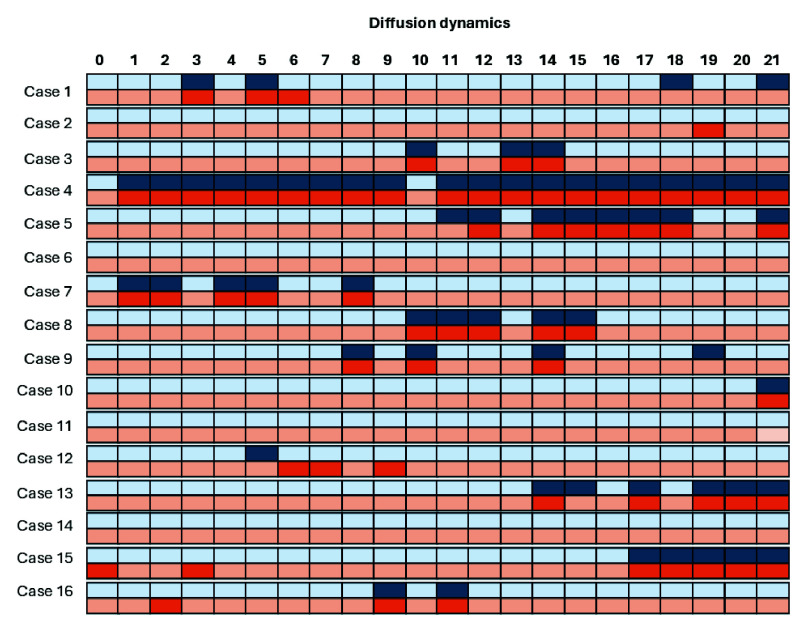
Evaluation of the real-time analyzed cases, depicting the assessment of the proposed automatic pipeline (orange) versus the dynamic-wise manual assessment by an expert observer (blue). Light colours indicate non-corrupted volumes and dark colours indicate corrupted volumes.

**Fig. 9.**
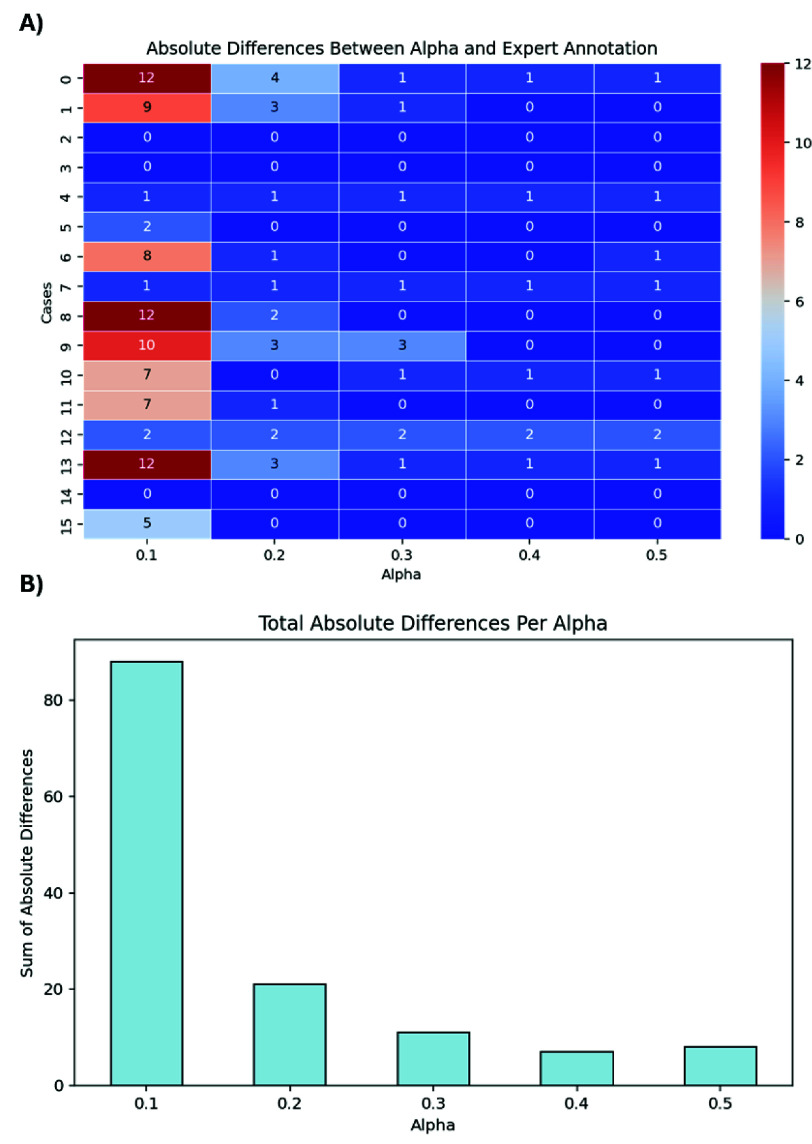
Quantitative results for the parameter $\alpha $. A) Heat map illustrating the differences between HERON motion detection and the expert annotation for $\alpha $ values ranging from 0.1 to 0.5. B) Total absolute differences across all considered datasets for the different choices for $\alpha $.

## IV. Discussion and Conclusion

### A. Summary

An automatic pipeline for real-time quality control of fetal brain dMRI was successfully deployed in vivo, using AI-enabled automatic planning of fetal brain dMRI, performing a quality check and triggering reacquisition where necessary. By seamlessly integrating real-time analysis with adaptive imaging protocols, this approach represents a significant leap forward in fetal dMRI methodology, establishing a strong foundation for achieving more precise, reliable and higher-quality diagnostic imaging for prenatal care.

### B. Comparison to the State of the Art

In contrast to the HERON approach, prior motion correction algorithms developed for neonatal and fetal diffusion MRI have been largely based on post-processing. Among these, Bastiani et al. [19] demonstrated the ability of FSL EDDY to address both intra- and inter-volume motion in neonatal data. This method combines corrections for susceptibility-induced distortions, eddy-current artifacts, and motion-related distortions in a single resampling step, offering a comprehensive solution for improving data quality. Christiaens et al. [28] introduced the SHARD Scattered Slice reconstruction method, which uses spherical harmonics and radial decomposition to reconstruct missing or corrupted slices in multi-shell neonatal diffusion MRI, while Deprez et al. [41] proposed a higher order spherical harmonic approach for fetal diffusion MRI with intensity correction, enhancing motion correction by modeling the diffusion signal more accurately. However, these methods are computationally intensive and are less effective in cases of pervasive motion or severe data corruption. In contrast, HERON offers real-time intra-volume motion correction with minimal latency. It can identify and re-acquire corrupted volumes within minutes of the initial acquisition, enhancing overall data quality. This approach prevents the accumulation of motion artifacts that can result in datasets unsuitable for post-processing correction, while also enabling more accurate inter-volume motion correction during subsequent post-processing steps.

Recent real-time motion correction techniques have shown significant effectiveness in reducing motion artifacts in adult brain dMRI. Some methods utilize optical tracking systems, such as the work by Berglund et al. [42], where an optical marker-less tracking system estimates and corrects rigid body motion of the head during the MRI acquisition. Other image-processing-based techniques such as Deep Learning based frameworks [43] or entropy-based methods [44] among others avoid the need for external devices, offering several advantages, especially in challenging clinical settings. However, implementing these techniques in fetal imaging is challenging due to the inability to use external tracking devices or the unpredictable and involuntary nature of fetal head motion, which can be more extensive than in neonatal or adult imaging due to the less constrained environment of the womb. The proposed pipeline, HERON, is able to provide real-time motion assessment by segmenting the fetal brain in each volume, detecting signal dropouts within the segmented volume suggesting motion-induced artifacts while also analyzing fetal brain volume changes resulting from inaccurate segmentations from motion-corrupted volumes.

### C. Strengths and Limitations

The presented method is capable of successfully deploying in real-time a pipeline for automatic planning, segmentation, data quality, and data reacquisition in fetal dMRI.

The ability to automatically segment the fetal brain immediately after data acquisition improves the efficiency of fetal brain volume assessment, offering a significant advantage over the time-consuming and labor-intensive process of manual segmentation and motion assessment. Furthermore, the large number of datasets employed for training and validation of the integrated network in HERON, which spans various field strengths, vendors, contrasts, GA, and pathologies, ensures broad generalizability. It is therefore expected that the proposed pipeline could be seamlessly applied to different scanners and field strengths with minimal to no adjustments.

HERON demonstrated successful real-time deployment with tools designed for low latency, allowing immediate responses during the acquisition. The results highlight its ability to assess dataset quality and reacquire volumes corrupted by intra-volume motion, which is challenging to correct with post-processing. The presented results show the ability of the proposed pipeline to significantly contribute to more reliable quantitative measurements in cases with low to moderate motion, where re-acquisition allowed complete correction of the corrupted volumes. For high-motion cases, the real-time information can trigger the decision to reacquire the sequence in another stage of the protocol when the fetus is more settled. Furthermore, the method was designed to perform with high sensitivity, hence prioritizing the identification of all potentially corrupted volumes at the expense of lower specificity, possibly at the expense of unnecessarily repeating some volumes.

Indicated in Figure 6, the major improvements are achieved with the replacement of the corrupted dynamics, denoising and registration alone in both the conventional and HERON pipeline led to only marginal qualitative improvements.

Despite its advantages, the presented method has certain limitations. The automatic planning step improves access to fetal imaging, particularly in less specialized centers, and enables reacquisition at any point in the protocol when necessary. Furthermore, while L2 norm calculation primarily detects affine motion of the brain and does not account for rotation, any rotational motion would be corrected through reacquisition, as landmark identification is independent.However, in the present study the repetition has always been acquired with the same planning in close temporal proximity to the original acquisition. Even minor shifts in the coordinates used to predict the correct axial radiological plane can highlight the need for post-scan registration to ensure the accuracy of quantitative maps. The volumetric registration technique used in this study is not specifically designed for diffusion data, making the registration of higher b-values more challenging in the presence of significant inter-volume motion, such as that occurring between re-acquisitions. As a result, while visual inspection suggests that HERON reduces corruption in the volumes compared to the original acquisition, inaccuracies in registration prevent the quantitative analysis from fully capturing this improvement particularly in cases where motion corruption is not severe. Future work on dedicated registration will improve this. Additionally, while the thresholding method employed to detect motion artifacts via signal dropouts is effective in most cases, it can occasionally misclassify slices. This is especially true when a slight tilt of the axial plane results in an oblique view of the longitudinal fissure, leading to false positives. In such instances, re-acquisition will continuously flag that volume as motion-corrupted. Moreover, while the presence of other artifacts such as geometric distortion and B1 inhomogeneities is generally lower at low field strengths, the system does not currently include automatic identification for these types of artifacts. As a result, any dataset directly affected by such artifacts will not be identified and thus not repeated in real-time. Finally, the current artifact detection step is based on a range of measures to identify different possible problems. A unified DL-based solution will be explored as an alternative once sufficient training data sets are available. These training data sets could concretely be labeled based on whether correction using post-processing was possible to guide the need for re-acquisition.

### D. Research Implications

Improving the quality of fetal dMRI with HERON opens up new possibilities for imaging in more challenging subjects, such as earlier gestational ages (GA). This is crucial for exploring early human brain development, including processes such as subplate emergence and neuronal migration [45]. Additionally, it supports advanced diffusion techniques like HARDI schemes and b-tensor encoding, which require a large number of volumes and are highly susceptible to motion artifacts. The ability to access motion information in real-time during the scan paves the way to explore new prospective intra- and inter-volume motion correction techniques avoiding data loss, particularly relevant in longitudinal studies to understand fetal brain development.The calculated L2 norm of the COM differences - while not used in the here presented work directly - provides the foundation for this.

### E. Clinical Implications

High data quality is of key importance, particularly for clinical fetal magnetic resonance imaging. The proposed method not only aids in identifying suboptimal scan quality in real-time, potentially minimizing the need for recalls, but also supports more accurate and reliable diagnoses of fetal brain conditions, such as agenesis of the corpus callosum and white matter abnormalities, by improving overall data quality. Furthermore, the real-time motion correction ability of HERON can improve the efficiency of clinical workflows, preventing the need for time-consuming reacquisition of entire dMRI datasets, and minimizing maternal stress.

### F. Future Steps

Future steps involve a more accurate automatic planning, applied de-novo for each repetitions, ensuring the exact position of the plane even with re-acquisition of the landmarks or maternal breaks. In addition, a more sophisticated inter-volume analysis and correction will be explored and added to HERON, including parallelized segmentation and analysis for further reduced latency, correcting both inter- and intra-volume motion in real time. Concretely, we will explore a machine learning approach for the corrupted volume detection step in the pipeline once enough data is available. Moreover, to improve the method’s efficiency, re-acquisition of only the directions affected by motion will be performed, minimizing redundant acquisitions and optimizing scan time, thereby enhancing overall workflow. Finally, deploying HERON in clinical fetal MRI cases, particularly those with neurological pathologies, will allow to study the ability of the method to facilitate more accurate and timely diagnosis, potentially contributing to clinical decisions and ultimately improving patient outcomes through more reliable imaging results.

## V. Conclusion

In conclusion, the HERON pipeline advances fetal dMRI by providing real-time motion correction and automatic data quality assessment, improving imaging accuracy and efficiency. Its ability to identify and reacquire corrupted volumes minimizes the need for manual intervention and enhances diagnostic reliability, especially in motion-prone fetal scans. HERON shows great potential to enhance both fetal neuroimaging research and clinical outcomes, particularly to help accelerate clinical adoption. Finally, the proposed real-time pipeline could prove significant benefit if adapted for other motion-prone populations such as unsedated pediatric subjects, patients suffering from motion disorders or participants with attention deficit hyperactivity disorder for example.

## References

[1] W. Zheng , “Diffusion-weighted MRI of the fetal brain in fetal growth restriction with maternal preeclampsia or gestational hypertension,” J. Magn. Reson. Imag., vol. 59, no. 4, pp. 1384–1393, Apr. 2024. 10.1002/jmri.28861 37315155

[2] J. W. Song, P. D. Mitchell, J. Kolasinski, P. E. Grant, A. M. Galaburda, and E. Takahashi, “Asymmetry of white matter pathways in developing human brains,” Cerebral Cortex, vol. 25, no. 9, pp. 2883–2893, Sep. 2015, doi: 10.1093/cercor/bhu084. 24812082 PMC4537435

[3] C. Jaimes , “In vivo characterization of emerging white matter microstructure in the fetal brain in the third trimester,” Hum. Brain Mapping, vol. 41, no. 12, pp. 3177–3185, Aug. 2020. [Online]. Available: https://onlinelibrary.wiley.com/doi/abs/10.1002/hbm.25006 10.1002/hbm.25006 PMC7375105 32374063

[4] M. C. Diogo , “Improved neurodevelopmental prognostication in isolated corpus callosal agenesis: Fetal magnetic resonance imaging-based scoring system,” Ultrasound Obstetrics Gynecol., vol. 58, no. 1, pp. 34–41, Jul. 2021. [Online]. Available: https://obgyn.onlinelibrary.wiley.com/doi/abs/10.1002/uog.22102 10.1002/uog.22102 PMC8362015 32484578

[5] R. Corroenne , “Quantitative fetal MRI with diffusion tensor imaging in cases with ’short’ corpus callosum,” Ultrasound Obstetrics Gynecol., vol. 63, no. 3, pp. 385–391, 2024. [Online]. Available: https://obgyn.onlinelibrary.wiley.com/doi/abs/10.1002/uog.27473 10.1002/uog.27473 37676105

[6] L. Witzani, P. C. Brugger, M. Hörmann, G. Kasprian, C. Csapone-Balassy, and D. Prayer, “Normal renal development investigated with fetal MRI,” Clin. Imag., vol. 30, no. 4, p. 300, Jul. 2006. 10.1016/j.ejrad.2005.11.027 16406436

[7] P. J. Slator , “Data-driven multi-contrast spectral microstructure imaging with InSpect: INtegrated SPECTral component estimation and mapping,” Med. Image Anal., vol. 71, Jul. 2021, Art. no. 102045. 10.1016/j.media.2021.102045 PMC8543043 33934005

[8] N. S. Higano , “Fetal lung development via quantitative biomarkers from diffusion MRI and histological validation in rhesus macaques,” J. Perinatology, vol. 42, no. 7, pp. 866–872, Oct. 2021. 10.1038/s41372-021-01236-x PMC9023595 34686834

[9] A. Jakab, R. Tuura, R. Kottke, C. J. Kellenberger, and I. Scheer, “Intra-voxel incoherent motion MRI of the living human foetus: Technique and test-retest repeatability,” Eur. Radiol. Experim., vol. 1, no. 1, Dec. 2017. 10.1186/s41747-017-0031-4 PMC5909359 29708192

[10] S. Wilson , “Development of human white matter pathways in utero over the second and third trimester,” Proc. Nat. Acad. Sci. USA, vol. 118, no. 20, May 2021, Art. no. 2023598118. [Online]. Available: https://www.pnas.org/doi/abs/10.1073/pnas.2023598118 10.1073/pnas.2023598118 PMC8157930 33972435

[11] S. Wilson , “Dynamic changes in subplate and cortical plate microstructure at the onset of cortical folding in vivo,” bioRxiv, Dec. 2024. [Online]. Available: https://www.biorxiv.org/content/early/2024/06/28/2023.10.16.562524

[12] C. Calixto , “Characterizing microstructural development in the fetal brain using diffusion MRI from 23 to 36 weeks of gestation,” Cerebral Cortex, vol. 34, no. 1, p. 409, Jan. 2024, doi: 10.1093/cercor/bhad409. PMC10793585 37948665

[13] R. Chen , “Deciphering the developmental order and microstructural patterns of early white matter pathways in a diffusion MRI based fetal brain atlas,” NeuroImage, vol. 264, Dec. 2022, Art. no. 119700. [Online]. Available: https://www.sciencedirect.com/science/article/pii/S1053811922008217 10.1016/j.neuroimage.2022.119700 36270621

[14] S. Khan , “Fetal brain growth portrayed by a spatiotemporal diffusion tensor MRI atlas computed from in utero images,” NeuroImage, vol. 185, pp. 593–608, Jan. 2019. [Online]. Available: https://www.sciencedirect.com/science/article/pii/S1053811918307286 30172006 10.1016/j.neuroimage.2018.08.030 PMC6289660

[15] L. Vasung, M. Raguz, I. Kostovic, and E. Takahashi, “Spatiotemporal relationship of brain pathways during human fetal development using high-angular resolution diffusion MR imaging and histology,” Frontiers Neurosci., vol. 11, Jul. 2017. [Online]. Available: https://api.semanticscholar.org/CorpusID 10.3389/fnins.2017.00348 PMC5504538 28744187

[16] J. D. Hooker , “Third-trimester in utero fetal brain diffusion tensor imaging fiber tractography: A prospective longitudinal characterization of normal white matter tract development,” Pediatric Radiol., vol. 50, no. 7, pp. 973–983, Jun. 2020, doi: 10.1007/s00247-020-04639-8. 32399686

[17] A. Lim, J. Lo, M. W. Wagner, B. Ertl-Wagner, and D. Sussman, “Motion artifact correction in fetal MRI based on a generative adversarial network method,” Biomed. Signal Process. Control, vol. 81, Mar. 2023, Art. no. 104484.

[18] H. Snoussi, D. Karimi, O. Afacan, M. Utkur, and A. Gholipour, “Advanced framework for fetal diffusion mri: Dynamic distortion and motion correction,” in Perinatal, Preterm and Paediatric Image Analysis. Cham, Switzerland: Springer, 2025, pp. 35–45. 10.1007/978-3-031-73260-7_4 PMC12013523 40265126

[19] M. Bastiani , “Automated processing pipeline for neonatal diffusion MRI in the developing human connectome project,” NeuroImage, vol. 185, pp. 750–763, Jan. 2019. 29852283 10.1016/j.neuroimage.2018.05.064 PMC6299258

[20] T. Sanchez , “FetMRQC: A robust quality control system for multicentric fetal brain MRI,” 2023, arXiv:2311.04780. 10.1016/j.media.2024.103282 39053168

[21] L. Vasung , “Cross-sectional observational study of typical in utero fetal movements using machine learning,” Develop. Neurosci., vol. 45, no. 3, pp. 105–114, Dec. 2022. 10.1159/000528757 PMC10233700 36538911

[22] J. Xu , “Fetal pose estimation in volumetric MRI using a 3D convolution neural network,” Med. Image Comput. Comput. Assist. Interv., vol. 11767, pp. 403–410, Oct. 2019. 32494782 10.1007/978-3-030-32251-9_44 PMC7267040

[23] H. Kebiri , “Deep learning microstructure estimation of developing brains from diffusion MRI: A newborn and fetal study,” Med. Image Anal., vol. 95, Jul. 2024, Art. no. 103186. [Online]. Available: https://www.sciencedirect.com/science/article/pii/S1361841524001117 10.1016/j.media.2024.103186 PMC11701975 38701657

[24] S. Neves Silva , “Real-time fetal brain tracking for functional fetal MRI,” Magn. Reson. Med., vol. 90, no. 6, pp. 2306–2320, Dec. 2023. 37465882 10.1002/mrm.29803 PMC10952752

[25] F. Zhang, W. M. Wells, and L. J. O’Donnell, “Deep diffusion MRI registration (DDMReg): A deep learning method for diffusion MRI registration,” IEEE Trans. Med. Imag., vol. 41, no. 6, pp. 1454–1467, Jun. 2022. 10.1109/TMI.2021.3139507 PMC9273049 34968177

[26] B. Marami, B. Scherrer, O. Afacan, B. Erem, S. K. Warfield, and A. Gholipour, “Motion-robust diffusion-weighted brain MRI reconstruction through slice-level registration-based motion tracking,” IEEE Trans. Med. Imag., vol. 35, no. 10, pp. 2258–2269, Oct. 2016. 10.1109/TMI.2016.2555244 PMC5108524 27834639

[27] S. Wang, D. J. Peterson, J. C. Gatenby, W. Li, T. J. Grabowski, and T. M. Madhyastha, “Evaluation of field map and nonlinear registration methods for correction of susceptibility artifacts in diffusion MRI,” Frontiers Neuroinform., vol. 11, Feb. 2017. 10.3389/fninf.2017.00017 PMC5318394 28270762

[28] D. Christiaens , “Scattered slice SHARD reconstruction for motion correction in multi-shell diffusion MRI,” NeuroImage, vol. 225, Jan. 2021, Art. no. 117437. [Online]. Available: https://www.sciencedirect.com/science/article/pii/S1053811920309228 10.1016/j.neuroimage.2020.117437 PMC7779423 33068713

[29] E. Caruyer, I. Aganj, C. Lenglet, G. Sapiro, and R. Deriche, “Motion detection in diffusion MRI via online ODF estimation,” Int. J. Biomed. Imag., vol. 2013, Feb. 2013, Art. no. 849363. 10.1155/2013/849363 PMC3594974 23509445

[30] M. Aksoy , “Real-time optical motion correction for diffusion tensor imaging,” Magn. Reson. Med., vol. 66, no. 2, pp. 366–378, Aug. 2011. [Online]. Available: https://onlinelibrary.wiley.com/doi/abs/10.1002/mrm.22787 21432898 10.1002/mrm.22787 PMC3139706

[31] T. Kober, R. Gruetter, and G. Krueger, “Prospective and retrospective motion correction in diffusion magnetic resonance imaging of the human brain,” NeuroImage, vol. 59, no. 1, pp. 389–398, Jan. 2012. [Online]. Available: https://www.sciencedirect.com/science/article/pii/S1053811911007646 21763773 10.1016/j.neuroimage.2011.07.004

[32] H. Chen , “High-resolution multi-shot diffusion-weighted MRI combining markerless prospective motion correction and locally low-rank constrained reconstruction,” Magn. Reson. Med., vol. 89, no. 2, pp. 605–619, Feb. 2023. [Online]. Available: https://onlinelibrary.wiley.com/doi/abs/10.1002/mrm.29468 36198013 10.1002/mrm.29468

[33] X. Liang , “Prospective motion detection and re-acquisition in diffusion MRI using a phase image–based method—Application to brain and tongue imaging,” Magn. Reson. Med., vol. 86, no. 2, pp. 725–737, Aug. 2021. 33665929 10.1002/mrm.28729 PMC9169515

[34] T. Benner, A. J. W. van der Kouwe, and A. G. Sorensen, “Diffusion imaging with prospective motion correction and reacquisition,” Magn. Reson. Med., vol. 66, no. 1, pp. 154–167, Jul. 2011. [Online]. Available: https://onlinelibrary.wiley.com/doi/abs/10.1002/mrm.22837 21695721 10.1002/mrm.22837 PMC3121006

[35] A. Bortolazzi , “Automatic assessment of fetal multi-echo diffusion weighted scans,” in Perinatal, Preterm and Paediatric Image Analysis, D. Link-Sourani, E. Abaci Turk, C. Macgowan, J. Hutter, A. Melbourne, and R. Licandro, Eds., Cham, Switzerland: Springer, Oct. 2024, pp. 82–93.

[36] K. Chow, P. Kellman, and H. Xue, “Prototyping image reconstruction and analysis with FIRE,” in Proc. 24th Annu. Sci. Sessions, Virtual Meeting (SCMR), Aug. 2021.

[37] M. S. Hansen and T. S. Sørensen, “Gadgetron: An open source framework for medical image reconstruction,” Magn. Reson. Med., vol. 69, no. 6, pp. 1768–1776, Jun. 2013. 22791598 10.1002/mrm.24389

[38] F. Isensee, P. F. Jaeger, S. A. A. Kohl, J. Petersen, and K. H. Maier-Hein, “NnU-net: A self-configuring method for deep learning-based biomedical image segmentation,” Nature Methods, vol. 18, no. 2, pp. 203–211, Dec. 2020. 33288961 10.1038/s41592-020-01008-z

[39] J.-D. Tournier , “MRtrix3: A fast, flexible and open software framework for medical image processing and visualisation,” NeuroImage, vol. 202, Nov. 2019, Art. no. 116137. [Online]. Available: https://www.sciencedirect.com/science/article/pii/S1053811919307281 10.1016/j.neuroimage.2019.116137 31473352

[40] R. H. J. Fick, D. Wassermann, and R. Deriche, “The dmipy toolbox: Diffusion MRI multi-compartment modeling and microstructure recovery made easy,” Frontiers Neuroinform., vol. 13, p. 64, Oct. 2019. 10.3389/fninf.2019.00064 PMC6803556 31680924

[41] M. Deprez , “Higher order spherical harmonics reconstruction of fetal diffusion MRI with intensity correction,” IEEE Trans. Med. Imag., vol. 39, no. 4, pp. 1104–1113, Apr. 2020. 10.1109/TMI.2019.2943565 31562073

[42] J. Berglund , “Prospective motion correction for diffusion weighted EPI of the brain using an optical markerless tracker,” Magn. Reson. Med., vol. 85, no. 3, pp. 1427–1440, Sep. 2020. 32989859 10.1002/mrm.28524 PMC7756594

[43] A. Ahmad, D. Parker, S. Dheer, Z. R. Samani, and R. Verma, “3D-QCNet—A pipeline for automated artifact detection in diffusion MRI images,” Computerized Med. Imag. Graph., vol. 103, Jan. 2023, Art. no. 102151. 10.1016/j.compmedimag.2022.102151 PMC10494975 36502764

[44] I. Oguz , “DTIPrep: Quality control of diffusion-weighted images,” Frontiers Neuroinform., vol. 8, Jun. 2014. 10.3389/fninf.2014.00004 PMC3906573 24523693

[45] I. Kostović, “The enigmatic fetal subplate compartment forms an early tangential cortical Nexus and provides the framework for construction of cortical connectivity,” Prog. Neurobiol., vol. 194, Nov. 2020, Art. no. 101883. [Online]. Available: https://www.sciencedirect.com/science/article/pii/S0301008220301386 10.1016/j.pneurobio.2020.101883 32659318

